# Molecular cloning and characterisation of chicken IL-18 binding protein

**DOI:** 10.1016/j.dci.2020.103850

**Published:** 2021-01

**Authors:** Mark S. Gibson, Angela Steyn, David Kealy, Bernd Kaspers, Mark S. Fife

**Affiliations:** a BioISI - Biosystems and Integrative Sciences Institute, Faculdade de Ciências da Universidade de Lisboa, Lisboa, Portugal; b The Pirbright Institute, Pirbright, Woking, UK; c York Biomedical Research Institute and Department of Biology, University of York, Heslington, York, UK; d Department of Veterinary Science, Ludwig-Maximilians-Universität, Munich, Germany; e Aviagen Ltd, Newbridge, UK

**Keywords:** IL-18 binding protein, Chicken, Intracellular, Bioactivity

## Abstract

The human IL-1 receptor family is comprised of 11 membrane bound or soluble receptors and the IL-18 binding protein (IL-18BP). These receptors are dispersed across seven genomic loci, with the majority at a single locus. Direct orthologues were identified in the chicken at conserved genomic loci; however, the IL-18BP remained absent from the first four builds of the chicken genome sequence. Subsequent assemblies identified the gene at a locus syntenic with mammals; however, these predicted sequences differed between genome builds and contained multiple errors. A partial IL-18BP-like sequence in the NCBI EST database was used to clone the full-length cDNA. A splice variant, which lacks the exon that encodes part of the signal peptide, was also cloned. Human IL-18BP is differentially spliced to produce a number of variants, which are all secreted. By contrast, the spliced chicken isoform was predicted to be intracellular, and we identified similar variants with the same exon missing in a limited number of divergent vertebrate species. Mammalian and viral IL-18BPs inhibit IL-18 activity by directly binding to this cytokine. Full-length and intracellular chicken IL-18BPs were equally effective at inhibiting IL-18-mediated IFN-γ release from an avian B-cell line. Analysis of the predicted chIL-18BP protein sequence revealed two crucial residues, which account for 50% of the binding affinity between human IL-18 and IL-18BP, are conserved in the chicken and a fowlpox-encoded homologue, fpv214. This suggests specific fowlpox viruses used in humans as a vaccine vector have the potential to dampen anti-viral host immune responses.

## Highlights

• We report the discovery and characterisation of IL-18 binding protein in the chicken.
• A spliced intracellular IL-18BP variant was cloned in the chicken.
• We identified other vertebrate species that also retain a predicted intracellular IL-18BP isoform.
• Both isoforms of chicken IL-18BP are bioactive.

## 1 Introduction

Interleukin-18 (IL-18) is an 18 kDa proinflammatory cytokine that plays important roles in the initiation and promotion of host defense and inflammation after infection or injury (Dinarello et al., 2013). Initially identified as an interferon-γ (IFN-γ) inducing factor, IL-18 was confirmed as a novel member of the IL-1 cytokine family based on its structural and functional similarity to other members of this family (Bazan et al., 1996). IL-18 is constitutively present in the circulation in the absence of an inflammatory stimulus. IL-18 activity is regulated *in vivo* by a negative feedback mechanism involving a naturally occurring inhibitor, the IL-18 binding protein (IL-18BP) (Hurgin et al., 2002).

IL-18BP binds to IL-18 with high affinity, preventing an interaction between IL-18 and its cell surface receptor (Kim et al., 2000). This results in the suppression of IFN-γ production and a reduction in T-helper type 1 (Th1) immune responses (Novick et al., 1999). Therefore, because IL-18 regulates Th1 cytokine responses that are essential for the cytotoxic T cell response (Novick et al., 1999), IL-18BP is regarded as a modulator of the earliest phases of the Th1 cytokine response. Structural and functional studies focusing on IL-18 and IL-18BPs revealed an essential binding site between a lysine (L) on IL-18 and a phenylalanine (F) on the IL-18BPs (Kim et al., 2000). IL-18BP isoforms, which differ primarily in their carboxyl termini and in their ability to inhibit the biological activity of IL-18, have been identified in mice and humans. IL-18BP has a fundamentally important role in host defence, with its absence shown to be the cause of fatal viral hepatitis (Belkaya et al., 2019).

Although IL-18BP is constitutively expressed in mononuclear cells, elevated expression is induced by IFN-γ. The human IL-18BP promoter is activated by two IFN-γ-induced transcription factors: IFN regulatory factor 1 (IRF-1) and CCAAT/enhanced binding protein B (C/EBPB) (Hurgin et al., 2002), which are synthesised *de novo* for this purpose. IFN-γ-mediated induction of IL-18BP provides a negative feedback mechanism to restrict IL-18-driven immune responses (Hurgin et al., 2002; Muhl et al., 2000).

Interleukin-37, formerly known as IL-1F7, is an anti-inflammatory cytokine of the IL-1 family (Nold et al., 2010) that forms a complex with IL-18BP resulting in a reduction in IL-18 activity (Bufler et al., 2002). A number of human studies have shown that Type I IFNs also induce IL-18BP expression in the context of inflammation, infection and cancer. IFN-α promotes anti-inflammatory effects via two cytokine families, namely TNF-α and the IL-1 family (Kaser et al., 2002). In patients with hepatitis C virus, IFN-α exerts anti-inflammatory effects *in vivo* through the induction of IL-18BP, thereby reducing free IL-18 (Kaser et al., 2002).

A common evolutionary feature of many cytokines is the presence of circulating soluble receptors that are generated from transmembrane receptors. Unlike other cytokine binding proteins, IL-18BP is not derived from the transmembrane IL-18R complex and has little homology to either receptor chain (IL-18Rα and IL-18Rβ) (Hurgin et al., 2002) which both possess three Ig-like domains whilst IL-18BP has only one. The similarities between IL-18BP and the inhibitory receptor for IL-1 (IL-1R2) were initially discussed (Novick et al., 1999) and later examined (Watanabe et al., 2005) with the conclusion that IL-18BP and IL-1R2 had a common ancestral gene. However, subsequent analysis convincingly described the conservation of exon/intron boundaries, protein structures and the amino acid (aa) sequence of a key binding site between human and viral IL-18BPs and another member of the IL-1R family, IL-1R9 (Booker and Grattan, 2017). IL-18BP is unequivocally more similar to IL-1R9, suggesting that this binding protein may have evolved from a duplication of IL-1R9.

Functional viral homologues of human IL-18BP (huIL-18BP) have been identified in all orthopoxviruses, including vaccinia virus, cowpox virus, and ectromelia virus (Krumm et al., 2008). These IL-18BPs are highly conserved amongst this group of viruses, and though they generally have low identity with the human homologue, they encode a highly conserved IL-18 binding region (Krumm et al., 2012). The tyrosine (Y97) and phenylalanine (F104) residues that dominate the binding affinity between huIL-18BP and huIL-18 are highly conserved amongst the diverse mammalian and poxvirus IL-18BPs with the exception of yatapoxvirus IL-18BP that has a threonine where the phenylalanine is normally found (Krumm et al., 2012). Viral IL-18BPs bind huIL-18 with high affinity, blocking its activity (Xiang and Moss, 1999, 2001).

Chicken IL-18 is synthesised as an inactive pro-peptide which is cleaved, most likely by caspase-1, after a conserved aspartic acid residue (Schneider et al., 2000). Its bioactivity differs from mammalian orthologues, as chIL-18 alone is sufficient to induce IFN-γ production from avian splenocytes, though this requires the presence of macrophages (Göbel et al., 2003). More specifically, chIL-18 induces the proliferation of, and IFN-γ release from, CD4^+^ T-cells (Göbel et al., 2003). Some inbred chicken lines, which express a different major histocompatability complex (MHC), require T-cell receptor (TCR) cross-linking to secrete IFN-γ in response to IL-18 stimulation (Göbel et al., 2003). Mammalian IL-18 requires IL-12 or TCR cross-linking to elicit the same effect.

Fowlpox virus genomes contain a number of sequences that appear to encode immune evasion proteins (Afonso et al., 2000; Laidlaw and Skinner, 2004), one of which, in FP9, is a putative vIL-18BP (Laidlaw and Skinner, 2004). It was identified after chIL-18, strongly suggesting the discovery of chicken IL-18BP was just a matter of time.

In this study we cloned and characterised a full-length and a spliced intracellular variant of chicken IL-18BP.

## 2 Materials and methods

### 2.1 In silico characterisation of chicken IL-18 binding protein

Using the huIL-18BP aa sequence, a TBLASTN search of the chicken NCBI EST database was carried out. This identified a single EST (Genbank I.D.: CD727808.1) with high nucleotide identity to the IL-18 binding domain of huIL-18BP. The predicted aa sequence of CD727808.1 was also aligned with the huIL-18BP aa sequence using clustal Ω (http://www.ebi.ac.uk/Tools/msa/clustalo/), with similarly high identity at this region.

An initial BLASTN query of v4.0 of the chicken genome (NCBI browser) was carried out using this putative chIL-18BP mRNA. The translated EST sequence was further analysed by TBLASTN against all of the eukaryotic genomes in the ENSEMBL genome browser and by BLASTP against the UniParc protein sequence database (http://www.uniprot.org/uniparc/) to further confirm its identity.

Following amplification and cloning, two novel chIL-18BP aa sequences were analysed for the presence of a signal peptide using the SignalP-5.0 server (http://www.cbs.dtu.dk/services/SignalP/) (Almagro Armenteros et al., 2019). As the initial multi-genome search used a truncated EST, both cloned cDNAs were translated and re-analysed by TBLASTN against all eukaryotic genomes in ENSEMBL.

Three predicted chIL-18BP aa sequences became available in January 2015 in v5.0 of the chicken genome sequence (NCBI). Clustal Ω was used to align these with the predicted aa sequences of the cDNAs we had cloned. Revised, predicted chIL-18BP aa sequences became available in May 2018 in the 6th (GRCg6a, annotation release 104) and current assembly of the chicken genome (NCBI).

All of the corresponding mRNA sequences were aligned using either clustalX v2.1 or Clustal Ω and any differences were examined base-by-base to attempt to understand how they arose.

Glycosylation of the chIL-18BP protein sequence was predicted using the NetNGlyc (http://www.cbs.dtu.dk/services/NetNGlyc/) and NetOGlyc (http://www.cbs.dtu.dk/services/NetOGlyc/) servers to determine potential N- and O-glycosylation sites.

Vertebrate IL-18BP aa sequences analysed in this study derived from UniProt: American Alligator_sv (UPI0007120815), American Alligator_fl (UPI00071175D4), Chinese Alligator_sv (UPI0006EAEE32), Chinese Alligator_fl (UPI0006EAE546), Alpine Marmot_sv1 (UPI000762B657), Alpine Marmot_sv2 (UPI0007626408), Alpine Marmot_fl (UPI00076290A5), Sheepshead Minnow_sv (UPI0007427538), Sheepshead Minnow_fl (UPI00074268BB), Turquoise Killifish_sv (UPI00077D6057), Turquoise Killifish_fl (UPI00077D311D).

Vertebrate IL-18BP aa sequences analysed in this study derived from NCBI: Bald Eagle (XP_010575074), Collared Flycatcher (XP_005038792), White Ruffed Manakin (XP_027519300), Zebra Finch (XP_030147569), American Crow (XP_017587224), Human (NP_001034749.1), Turkeypox virus (YP_009177177), fpv073 (AF198100_64), fpv214 (AAF44558.1).

### 2.2 Chicken tissues, cells and nucleic acid templates

For the cDNA cloning, four different RNA templates were used. These were: Infectious bursal disease virus (IBDV)-infected bursa of Fabricius from resistant Line 6_1_ birds at 4 days post infection (dpi); bone marrow derived macrophage (BMDM) from line 7_2_ chickens stimulated for 4 h with 200 ng/ml LPS; *Salmonella typhimurium*-infected Rhode Island Red chicken splenocytes from 3 dpi; and splenocytes from Rhode Island Red chickens. All templates were generated as described (Gibson et al., 2012).

RNA was isolated from peripheral blood from eight lines of inbred White leghorn chickens (6_1_, 7_2_, 15I, C, N, O, P and W) and two relatively outbred chicken lines (Rhode Island Red (RIR) and Brown Leghorn (BrL)). Genomic DNA was isolated from peripheral blood from a RIR chicken. All birds were bred and sustained at IAH, Compton, UK. All RNA samples were isolated and reverse transcribed as described (Gibson et al., 2012).

### 2.3 Cloning and sequencing the IL-18BP cDNA

To determine the full-length IL-18BP cDNA, rapid amplification of cDNA ends (RACE) was carried out with the SMARTer RACE kit (Clontech, CA, USA) using primers designed against the EST (CD727808.1). Primer sequences: 3′ RACE primer: TGACGCTGCTCTACTGGTTGGGAAA; 5′ RACE primer: GGTGCTCAGGTCCTGGAAGCTGAAT. Four RNA templates were used to clone the cDNAs and were selected according to the expression profile and biology of mammalian IL-18BP. They were: splenocytes, IBDV-infected bursal cells, Salmonella *Typhimurium*-infected splenocytes and LPS-stimulated bone marrow-derived macrophages. All RNA templates were derived from previous studies in our lab. Thermal cycling conditions were those specified in the manufacturers protocol. RACE PCR products of interest were purified from agarose gels using the QIAquick gel extraction kit (Qiagen) and sequenced by the chain termination method. Analysis of the RACE sequences identified the start and stop codons allowing a further pair of primers to be designed to amplify the coding DNA sequence (CDS). Primer sequences: Forward: ATGGCTCCGTGTCCCCGCTC; Reverse: TCAGCCCATTCCCCCCCCTT. Using Rhode Island Red (RIR) splenocyte cDNA, two IL-18BP coding region sequences were PCR amplified with 0.625 U Go*Taq* DNA polymerase (Promega, Southampton, UK) in a final volume of 25 μl, and TA cloned into the pGEM-T easy vector (Promega) following manufacturers instructions. Thermal cycling conditions for the PCR were: 95 °C for 5 min; 30 cycles of 95 °C for 30 s, 59 °C for 30 s, 72 °C for 1 min. Clones were bi-directionally sequenced by the chain termination method using T7 and SP6 vector primers. The complete CDS of both IL-18BP variants from a RIR chicken was submitted to the European Nucleotide Archive (http://www.ebi.ac.uk/ena) with the accession numbers: LR812478 & LR812479. The full-length CDS for Brown leghorn (BrL) and lines W, 0, 6_1_, 7_2_, 15I, C, N, and P were also submitted to the same repository with the accession numbers: LR812480-LR812488.

Further primers were designed to directionally clone both IL-18BP sequences into the His-tagged pHLSec expression vector (kindly provided by James Birch, The Pirbright Institute; vector details are available (Aricescu et al., 2006)). Two forward primers, containing an *Age*I restriction site for full-length and splice variant cDNAs, and a universal reverse primer containing a *Kpn*I site were used. Start and stop codons were omitted from these primer sequences as they are provided by pHLSec. As this construct also encodes a signal peptide, the forward primer for the full-length cDNA was designed to bind IL-18BP immediately after this feature. The splice variant forward primer spans the nucleotide positions where splicing occurs. Primer sequences: Forward (full-length): GAGAGAaccggtATGGCCCTGCAGCCCCCCCG; Forward (splice variant): GAGAGAaccggtGCTCCGTGTCCCCGCTCCGACGCCATGGC; universal reverse primer: GAGAGAggtaccGCCCATTCCCCCCCCTTCGT. A further modified construct was created containing the entire chIL-18BP CDS. To do this, the vector was cut at a *Hind*III site, upstream from the multiple cloning site, to excise the construct signal sequence. A forward primer containing a *Hind*III sequence was designed to insert the full-length cDNA at this location. Sequence: GAGAGAaagcttGCCACCATGGCTCCGTGTCCCCGCTCCGA. This primer was paired with the same universal reverse primer. All primers were acquired from Sigma-Aldrich, Dorset, UK.

Using genomic DNA from a RIR chicken, the full IL-18BP gene was amplified by PCR and sequenced by the chain termination method. The complete genomic sequence was submitted to Genbank (https://www.ncbi.nlm.nih.gov/genbank/) with the accession number: MT635599.

### 2.4 Expression of recombinant protein in HEK293T cells

HEK293T cells were routinely cultured in DMEM with 10% FCS, 1% 200 mM l-glutamine, 1% non-essential amino acids and 0.1% penicillin/streptomycin (all supplied by Sigma) at 37 °C, 5% CO_2_. To transfect, 50 μg endotoxin-free plasmid DNA (pHLSec-IL-18BP) was mixed with 5 ml serum-free DMEM. To this, 75 μl of 1 mg/ml polyethylenimine (PEI) (Sigma) was added, mixed, and then incubated at room temperature for 10 min to allow the DNA and PEI to associate. During this incubation, growth medium was removed from the HEK293T cultures and replaced with DMEM containing 2% FCS. The DNA-PEI mixture was subsequently added to the cultures. Media was harvested at 4 days post-transfection; cultures were replenished with fresh medium and incubated for a further 4 days to produce a second batch of conditioned medium (CM). This was pooled with media from the first collection and centrifuged at 2000×*g* for 15 min at 4 °C to remove impurities and residual cells. Proteins were purified under native conditions using HisTrap FF columns (GE healthcare, Bucks, UK) and concentrated using Amicon Ultra-15 Centrifugal Filters 10K (Merck Millipore, Herts, UK). Proteins were resolved by SDS-PAGE using 4–20% Mini-PROTEAN gels (BIO-RAD, Hemel Hempstead, UK) and detected by Western blotting using the PentaHIS monoclonal primary antibody (Qiagen, Crawley, UK) and a goat anti-mouse secondary antibody (926–68070; Li-Cor Biosciences, Cambs, UK). The signal was detected by chemiluminescence using Amersham ECL products (GE healthcare). Pure IL-18BP was quantified using a Qubit 2.0 Fluorometer and the Qubit protein assay kit (both ThermoFisher Scientific, UK).

### 2.5 Deglycosylation of recombinant chIL-18BP protein

40 μl of FL or SV chIL-18BP concentrate was incubated with 5 μl 10× G7 reaction buffer (New England Biolabs (NEB), Ipswich, MA, USA) and 5 μl deglycosylation enzyme cocktail (P6039S, NEB) at 37 °C for 5 h. Deglycosylated proteins were resolved by SDS-PAGE and detected by Western blotting as described above. Precision plus protein™ WesternC™ blotting standards (BIO-RAD) were used to confirm the molecular weight of the applied recombinant proteins.

### 2.6 Characterization of IL-18BP bioactivity

B19-2D8 cells (Puehler et al., 2003) were seeded (2.5 × 10^5^ cells/well) into a flat bottom 96-well plate, then cultured at 41 °C for 2 h. During this incubation, rchIL-18BP was mixed with recombinant chicken IL-18 (ab87525; Abcam, Cambs, UK). A two-fold dilution series of chIL-18BP was prepared in triplicate in the range 16–1000 ng/ml 250 ng/ml chIL-18 was added to each concentration of chIL-18BP, then incubated at 41 °C for 1 h. Next, pre-incubated cytokines were added to the B19-2D8 cells and cultured for 48 h at 41 °C. Three concentrations of chIL-18 (15.62, 31.25, 62.5 ng/ml) were used as positive controls, with 250 ng/ml IL-18BP or IL-18BP_SV as negative controls. During the final 4 h of the assay, aliquots of each of the three [chIL-18] were removed and incubated with a 1:20 dilution of anti-chIFNγ polyclonal rabbit serum at 41 °C.

To determine the inhibition of IL-18-mediated IFNγ production by IL-18BP, a macrophage activation assay (Göbel et al., 2003; Schneider et al., 2000) was used. Nitric oxide is produced by chicken macrophages in response to pathogen invasion. The HD11 macrophage cell line produces nitrites following stimulation with chIFNγ (Siatskas et al., 1997). HD11 cells (1 × 10^5^ cells/well) were cultured with supernatants from B19-2D8 cells stimulated with either IL-18 (±anti-chIFNγ), or IL-18 + IL-18BP, or IL-18BP, as described above, or media alone at 41 °C for 24 h. Culture medium was tested for the presence of nitrites using a Griess Reagent System assay kit (Promega). Absorbance was quantified using a MultiskanGo plate reader (ThermoFisher Scientific).

### 2.7 Statistics

Statistical analyses were carried out using the Mann–Whitney test within the Prism 8 software package, version 8.4.1 (www.graphpad.com). Tests compared the chIL-18 + rchIL-18BP treatment groups to the chIL-18 only treatment within the B19-2D8 bioassay. Statistical significance was determined as *p < 0.05 (significant) up to ****p < 0.0001 (highly significant).

## 3 Results

### 3.1 Molecular cloning of two chicken IL-18 binding protein isoforms

A previous study (Watanabe et al., 2005) identified a truncated chicken expressed sequence tag (Accession: CD727808), which was predicted to contain an IL-18 binding domain, however, no further validation of this sequence was described. As the IL-18 binding protein (IL-18BP) was the only member of the chicken IL-1 receptor (chIL-1R) family missing from the chicken genome assembly, we sought to clone and characterise this cytokine to confirm its existence in an avian species. This transcript lacked a start codon and as EST sequence quality is typically poor, primers were designed to amplify the full-length cDNA by rapid amplification of cDNA ends (RACE). PCR products were synthesised from four different RNA templates, namely infectious bursal disease virus (IBDV)-infected bursa, LPS and CD40L-stimulated bone marrow-derived macrophage (BMDM), Salmonella *Typhimurium*-infected splenocyte and unstimulated splenocyte in order to capture the entire transcript. All 3′ RACE PCRs produced a single band of 519 bp in length (Supp Figure 1). Based on the length of CD727808, mammalian IL-18BP CDS sequences and the position of our primers, the 5′ RACE amplicon was predicted to be ≤ 500 bp. Agarose gel electrophoresis of the 5′ RACE products identified two prominent bands under 500 bp in the splenocyte and BMDM template PCRs (Supp Figure 1). Products were cloned from the splenocyte template and sequenced, confirming they span 482 bp and 419 bp in length, contain predicted start codons and share 100% sequence identity in overlapping regions. The smaller 5′ end fragment lacked a 63 bp contiguous region present in the larger product, suggesting this gene may encode splice variants similar to those identified in mammals. The same RACE PCRs using templates from IBDV and S. *Typhimurium*-challenged cells produced identical 3′ RACE bands, however, 5′ end amplicons were smeared or weak and therefore not cloned. Sequence analysis of overlapping RACE PCR products confirmed the full-length CDS spans 459 bp. Further primers were used to amplify this cDNA from the same original templates. Gel electrophoresis of the products again identified an additional smaller band (**data not shown**). PCR products were gel purified, cloned and sequenced, confirming the existence of a distinct 396 bp splice variant (SV) lacking a contiguous 63 bp region present in the full-length CDS. This splice variant of chIL-18BP (chIL-18BP_sv) lacks a region found in the full-length sequence towards the 5′ end. The function of IL-18BP relies on secretion of this cytokine to sequester IL-18. Both chicken variants were therefore analysed for the presence of a signal peptide using SignalP. A 30 aa signal peptide was identified in the full-length aa, however, the truncated chIL-18BP_sv does not contain this feature and would therefore be predicted to remain intracellular (Supp Figure 2). All the human IL-18BP variants present in the ENSEMBL genome browser possess a predicted signal sequence (**data not shown**).

### 3.2 Bioinformatic analysis of chIL-18BP sequences

In the course of this study two improved genome builds (Gallus_gallus-5.0 and GRCg6a) became available, which confirmed the IL-18BP locus in the chicken. Transcripts mapped to the chicken genome build identify three aa variants of this gene, all of which are predicted, and are annotated in the NCBI browser. The shortest of these, a 721 bp transcript (NCBI accession: XM_015280902.2) derived from RNA-Seq data, encodes a predicted 131-residue amino acid. This protein has 100% sequence identity with the splice variant (chIL-18BP_sv) we cloned, independently verifying its existence.

**Fig. 1:**
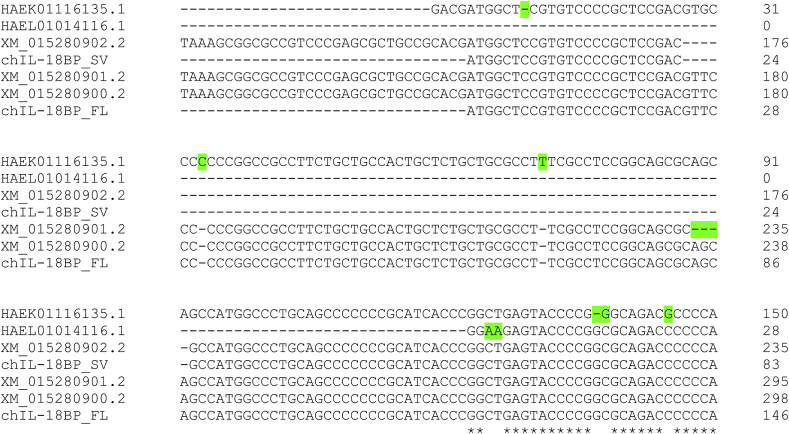

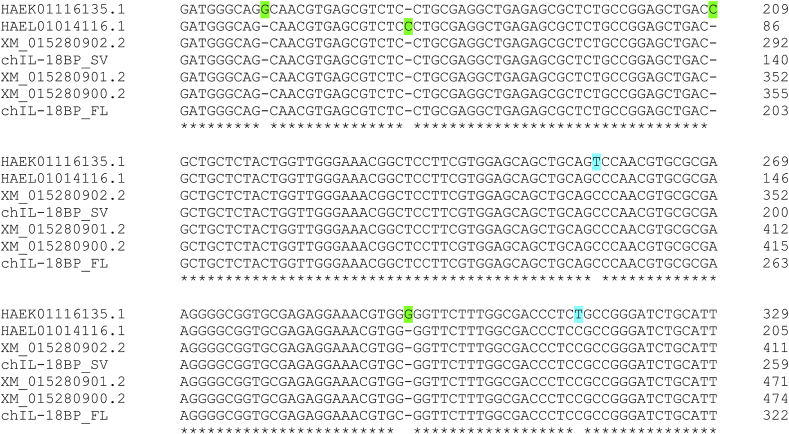

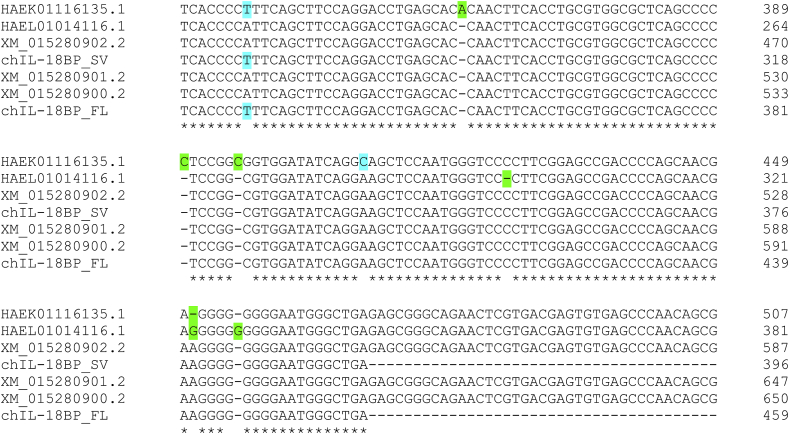
Alignment of chIL-18BP mRNA sequences. The full-length and splice variant CDS of cDNAs cloned in this study from RIR splenocytes were aligned with mRNAs listed in the NCBI Gene record using ClustalX. Three of these mRNAs are the current reference sequences in the RJF Gallus gallus GRCg6a genome assembly (X1, XM_015280900.2; X2, XM_015280901.2; X3, XM_015280900.2). Two other mRNAs (HAEK01116135.1, HAEL01014116.1) are listed as related sequences, and were derived from J-Line brain and ISA Brown whole embryo, respectively. Missing or incorrectly inserted nucleotides are highlighted in green. SNPs, either probable or confirmed in the cDNAs cloned in this study, are highlighted in blue. Asterisks beneath the alignment indicate the nucleotide is conserved at that position in all sequences.

The two additional predicted chIL-18BP transcripts (GRCg6a accession numbers: transcript variant X1, XM_015280900.2; transcript variant X2, XM_015280901.2) are present in NCBI, though after studying both Gallus_gallus-5.0 and GRCg6a builds, we identified what we believe are a small number of sequencing errors. Both encode the full-length aa, however, in Gallus_gallus-5.0, these mRNAs (variant X1, XM_015280900.1; variant X2, XM_015280901.1), when translated, encode predicted proteins of 221 and 220 aa in length and contain unusual aa sequence features. Both are much longer than mammalian and other non-mammalian IL-18BP proteins identifiable in UniProt; both contain a string of eleven consecutive glutamic acid residues following the predicted start codon and both contain an unusually long predicted signal sequence. To understand these anomalies, we aligned the cDNAs and 5′RACE products we cloned, all three predicted mRNAs and the original EST (CD727808). This identified a missing cytosine base at nt position 31 of the CDS of predicted transcript variants X1 and X2, which is present in all the other sequences. The absence of this nucleotide changes the reading frame in predicted variants X1 and X2 and consequently the entire aa sequence in the 5′ direction from this position. This demonstrated the RNA-Seq data and therefore the Gallus_gallus-5.0 genome sequence were incorrect at this position. Updated predicted sequences are now available in the GRCg6a build for these transcript variants (X1, XM_015280900.2; X2, XM_015280901.2). In both sequences, this error has now been corrected. The X1 variant is 784 nt in length and encodes a predicted protein (XP_015136386.2) of 152 residues, which has 100% identity with the translated full-length cDNA we cloned. The X2 variant, however, is 3 nt shorter than X1 and lacks this codon at positions 235–237 of the CDS (Fig. 1). Although this missing codon is in frame, the predicted amino acid sequence (XP_015136387.2) is therefore incorrect.

In the NCBI Gene record for chicken IL-18BP, two mRNA sequences derived from PacBio sequencing of a transcript library, are listed (GenBank I.Ds: HAEL01014116.1 and HAEK01116135.1). Aligning these sequences with our cDNAs and the transcript variants annotated in the NCBI genome browser (Fig. 1) reveals both contain a number of errors, causing frameshifts and consequently altering their predicted amino acid sequences. We are confident our CDS and aa sequences are correct based on the number of independent clones we PCR amplified and Sanger sequenced as well as alignments with the current GRCg6a build and other avian IL-18BP protein sequences (Supp.Fig 3).

### 3.3 A truncated intracellular IL-18BP isoform is conserved between divergent classes of vertebrates

**Fig. 2:**
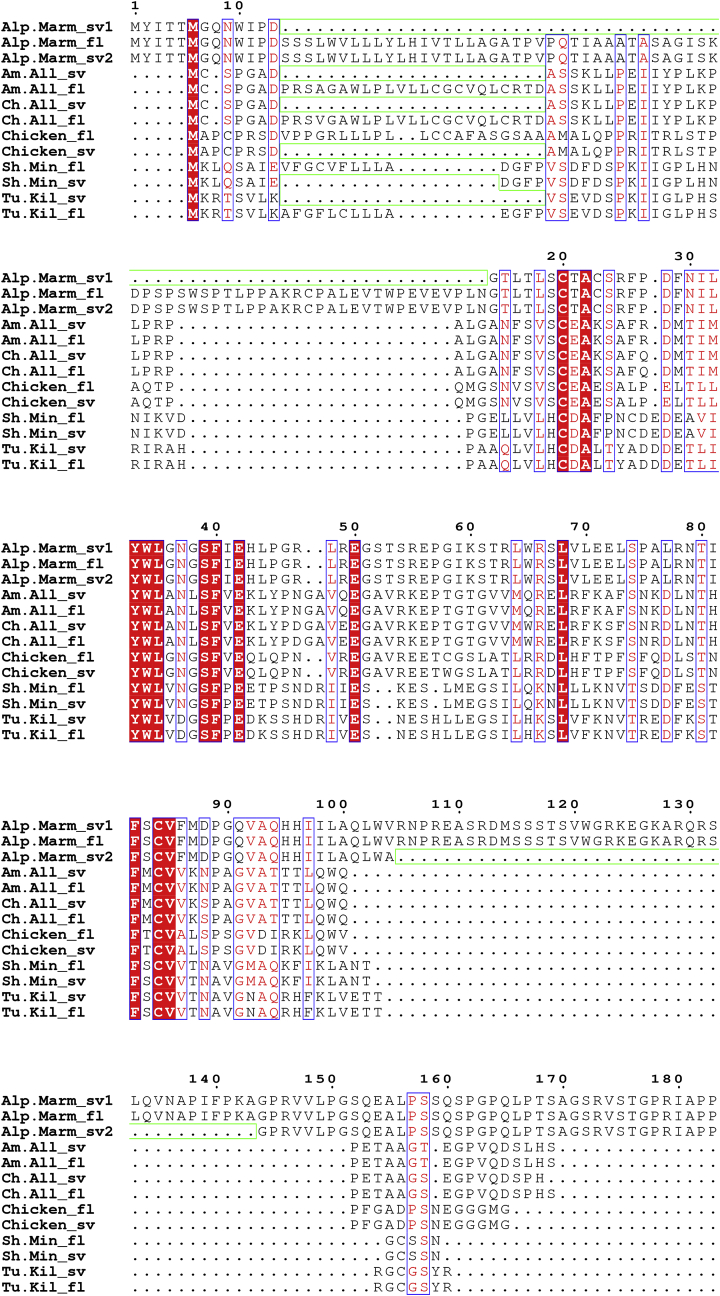
Alignment of spliced intracellular IL-18BP amino acid sequences. ClustalX was used to align the full-length and splice variant IL-18BP aa sequences of a group of vertebrate species. These sequences were originally derived from a BLASTP search of the UniProt database (UniParc sequence archive) using the full-length chIL-18BP aa sequence. The ALN file derived from the Clustal X alignment was submitted to ESPript 3.0 to create the figure. Residues conserved in all sequences have red background shading and are boxed. Residues conserved in some of the sequences have red letters and are boxed. The regions where residues are missing in the sv sequences are outlined with a green box. Alp. Marm = Alpine Marmot; Am.All = American Alligator; Ch. All = Chinese Alligator; Sh. Min = Sheepshead Minnow; Tu. Kil = Turquoise Killifish.

The BLASTP analysis of UniProt uncovered more than one hit from many of the species identified. These sequences were examined using Clustal to identify whether different isoforms, similar to those we cloned in the chicken, were present in other species. We found a limited number of species express a spliced IL-18BP, with each encoding an isoform lacking the second exon that contains some of the signal peptide (Fig. 2). Remarkably, this group is comprised of two reptiles (American Alligator, Chinese Alligator), one mammal (Alpine Marmot) and two fish species (Sheepshead Minnow, Turquoise Killifish), which, along with the chicken_sv sequence, suggests a widely conserved mechanism may exist to create an intracellular IL-18BP isoform. SignalP analysis confirms that in all six species this isoform lacks a recognisable signal peptide (**data not shown**). As the genome sequence of all six species is available in NCBI, the map viewer tool was used to confirm each splice variant lacks the entire second exon, with the exception of the Sheepshead Minnow sequence (isoform X2), which retains 12 nucleotides from this CDS region. It is possible that other species also encode intracellular IL-18BP isoforms, however, due to a lack of data, this remains unknown. Although human IL-18BP encodes many splice variants (Kim et al., 2000), none of these lack the signal sequence required for secretion.

### 3.4 Inbred lines of chickens exhibit limited polymorphism

The huIL-18BP CDS contains 135 polymorphic positions, of which 98 are missense mutations. From the total number of SNPs, eight are positioned within the IL-18 binding domain, a sixteen aa region beginning at F95. Seven of these residues directly contact IL-18 and one of them, F95C, is polymorphic, which may affect binding affinity. We therefore sought to determine the level of polymorphism in the chicken IL-18BP CDS using eight inbred and two relatively outbred lines, which differ in their resistance to three common viral pathogens. Primers that captured the full-length CDS were used to PCR amplify the transcript in all ten lines. Sequence analysis revealed only nine nucleotide positions from the full-length CDS differed between the lines. Lines 6, 7, 15, C, N and P are identical, however, five of these nine SNPs are synonymous and present in Line W (1), Line 0 (1), RIR (1) and BrL (2) birds. A further four non-synonymous SNPs were identified in Line W (P144L), RIR (W96C) and BrL (T47P, L102P) birds. Based on a comprehensive review of six studies examining human or viral IL-18BP, either binding or inhibiting huIL-18 (Booker and Grattan, 2017), none of the polymorphic residues in chIL-18BP are at conserved positions that affect binding. T47P in BrL birds is conserved with a residue used by Yatapox vírus (P32) to homodimerise, however, other viruses and mammalian IL-18BPs have a different aa at this position. Furthermore, mammalian IL-18BPs exist as monomers suggesting this conserved proline in chIL-18BP is unlikely to retain the same function as in Yatapox IL-18BP.

The huIL-18BP gene is highly polymorphic in both untranslated regions (UTR) of exon sequence and within all of the introns. To establish how polymorphic these regions may also be in the chicken, we used a single pair of primers to PCR amplify the full genomic sequence in the RIR breed. A BLASTn search with this RIR sequence against the current GRCg6a genome assembly revealed a pairwise sequence identity of 99.2%, with three indels and eight SNPs. Four of the SNPs are intronic, with two of the four exonic SNPs present in UTRs (data not shown). Similar to the CDS, the full chIL-18BP genomic sequence also has very limited polymorphism.

### 3.5 Avian poxvirus IL-18BP homologues possess conserved IL-18 binding residues

**Fig. 3:**
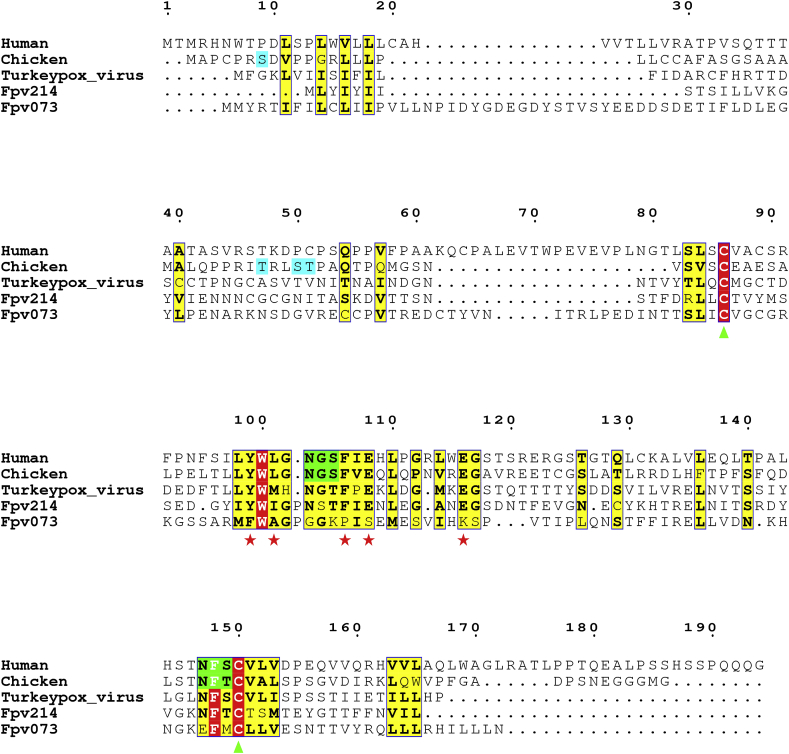
Amino acid alignment of chicken, human and fowlpox virus IL-18BP sequences. ClustalX was used to align the full-length chicken and human IL-18BP aa sequences with Turkeypox virus and fowlpox virus-encoded homologues. The ALN file derived from this alignment was submitted to ESPript 3.0 to create the figure. Residues conserved in all sequences have red background shading and are boxed. Residues conserved in some of the sequences have yellow background shading and are boxed. Predicted N-linked glycosylation sites shared by human and chicken sequences have green background shading. Four predicted O-linked residues (S7, T39, S42, T43) in the chicken sequence have blue background shading. Two cysteine residues, conserved in all sequences, and likely to form an intrachain disulphide bond are marked with a green triangle beneath the alignment. Red stars beneath the alignment denote the residues that site A of huIL-18 uses to bind to huIL-18BP (Krumm et al., 2008).

Within the chIL-18BP Ig domain, the region exhibiting the highest identity (68%) with the human homologue is the IL-18 binding motif (Fig. 3). Two previous studies (Afonso et al., 2000; Skinner et al., 2005) identified predicted fowlpox virus (fpv)-encoded homologues of chIL-18BP (fpv073 and fpv214, respectively) and both sequences also demonstrate a degree of aa identity with mammalian orthologues at this functional domain (Fig. 3). As shown in the figure, both fowlpox viral proteins have very low identity with the native sequence(s) outside of this motif. Mutagenesis of this region in huIL-18BP identified five essential residues used to bind huIL-18. All of them are conserved in chIL-18BP, whilst four are conserved in fpv214. A fifth residue (isoleucine) in fpv214 is biochemically very similar to the native human aa (leucine) (Fig. 3). By contrast, fpv073 has no identity with chicken or human IL-18BP at any of these positions. Four of these five residues are also conserved between Turkey poxvirus and the huIL-18BP homologue (Fig. 3). These data strongly suggest fpv214 will exhibit IL-18BP bioactivity in the chicken, but fpv073 will not. HuIL-18 has three sites (A, B & C) that IL-18BP binds to, however, site A, containing these five key positions, is considered the most vital (Krumm et al., 2008). Just two of these aa, Y97 and F104 account for 50% of the “free energy” required for binding (Xiang and Moss, 2001). If these two residues, which are conserved in chIL-18BP and fpv214, are functional binding epitopes in avipoxviruses, it is conceivable that some fpv-vectored vaccines may inhibit huIL-18 using their expressed vIL-18BP, reducing the effectiveness of human anti-viral immune responses.

### 3.6 Full length and spliced chIL-18BP isoforms are bioactive

**Fig. 4:**
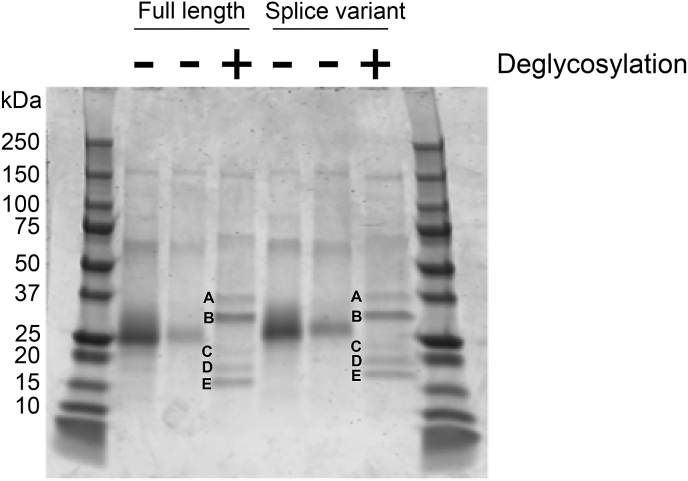
Recombinant full-length and splice variant chIL-18BP proteins are glycosylated. Aliquots of concentrated FL or SV chIL-18BP proteins were incubated with G7 reaction buffer (NEB) and deglycosylation enzyme cocktail at 37 °C for 5 h. Deglycosylated proteins were resolved by SDS-PAGE and detected by Western blotting. Untreated proteins are in the lanes with a minus symbol above. In these lanes, rchIL-18BP_FL has a molecular weight (MW) = 27 kDa, and the rchIL-18BP_SV MW = 28 kDa. Deglycosylated proteins are in the lanes with a plus symbol above. In these two lanes; A = neuraminidase, B = Peptide-N-Glycosidase F (PNGase F), C & D = partially deglycosylated forms of chIL-18BP, E = fully deglycosylated forms of chIL-18BP.

Recombinant full-length and splice variant (SV) isoforms of chIL-18BP were expressed in HEK293T cells and purified. Western blot analysis revealed these proteins have a molecular weight of 27 kDa and 28 kDa, respectively (Fig. 4). This is considerably more than the predicted 14.5 kDa and 15.3 kDa, suggesting post-translational modification. Human IL-18BP is predicted to be glycosylated, therefore we analysed both sequences for residues that facilitate this feature. Three N-linked glycosylation sites, at residues 54, 76 and 120 of the full-length sequence; and four O-linked residues S7, T39, S42, T43 were predicted (Fig. 3). Treatment with a de-glycosylating enzyme cocktail produced three smaller protein species (labelled C, D & E) for each isoform, with the smallest (E) around 16 kDa and 17 kDa for FL and SV, respectively (Fig. 4). The two other digested species for each isoform (C & D) had higher molecular weights, suggesting they were partially de-glycosylated.

**Fig. 5:**
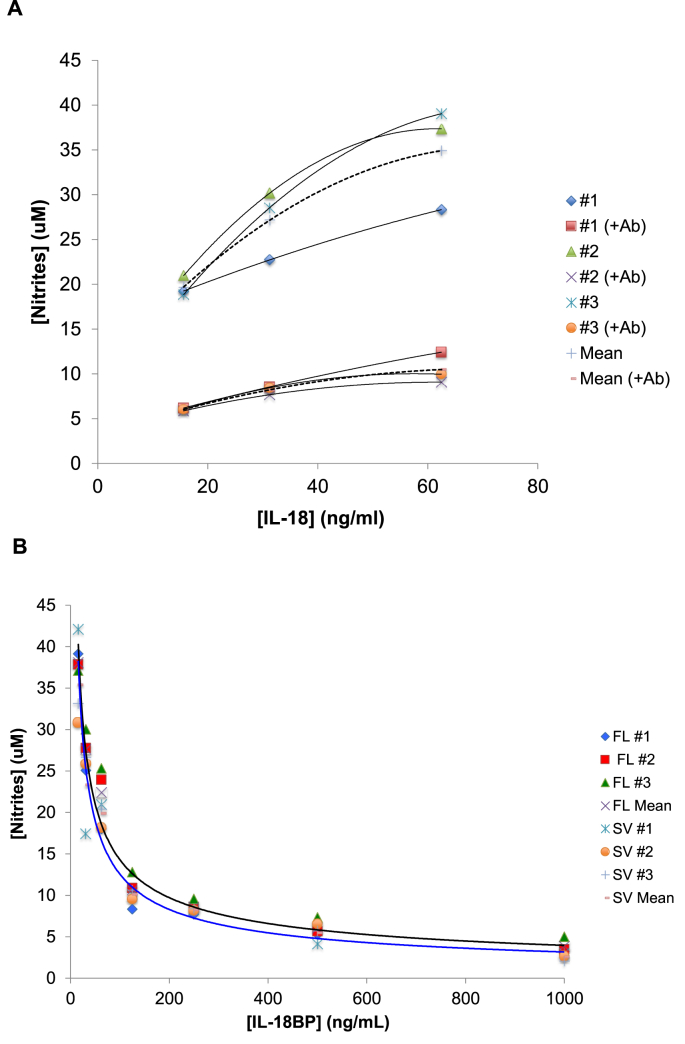
Inhibition of chIL-18-induced IFN-γ production by chIL-18BP. (A) B19-2D8 cells were stimulated with three concentrations of recombinant chIL-18 for 48 h. Supernatants were harvested and incubated with either a 1/20 dilution of polyclonal anti–IFN–γ antibody (Puehler et al., 2003) or untreated for 2 h at 41 °C. Next, HD11 cells were incubated with these B19-2D8 cell supernatants for 48 h at 41 °C. HD11 cell supernatants were then tested for the presence of nitrites using the Griess assay. Nitrite concentrations from three independent assays and the mean values are presented. (B) A doubling dilution series of FL and SV rchIL-18BP (1000–15.625 ng/ml) was made and each concentration was pre-incubated with 250 ng/ml rchIL-18 for 1 h at 41 °C. Next, these chIL-18BP:chIL-18 complexes, or 250 ng/ml rchIL-18 (positive control) or 250 ng/ml of either FL or SV chIL-18BP (negative controls) were added to B19-2D8 cells and incubated for 48 h at 41 °C. HD11 cells were incubated with these B19-2D8 cell supernatants for 48 h at 41 °C. HD11 cell supernatants were then tested for the presence of nitrites using the Griess assay. Nitrite concentrations from three independent assays and the mean values are presented. *p < 0.05, **p < 0.01, ***p < 0.001, ****p < 0.0001.

In mammals, IL-18 + IL-12 or a low dose of LPS or TNF-α, induces IFN-γ production (Novick et al., 1999). By contrast, chIL-18 alone readily induces IFN-γ production in chicken splenocytes (Schneider et al., 2000). This effect can be assayed indirectly by stimulating an avian macrophage cell line, termed HD11, with IFN-γ-containing supernatants to induce nitrite production, as previously described (Göbel et al., 2003; Schneider et al., 2000). In addition to this, a retro-virally transformed avian B-cell line, named B19-2D8, rapidly releases IFN-γ from cytoplasmic stores upon stimulation with IL-18 (Puehler et al., 2003). The ability of purified chIL-18BP to bind to and inhibit IL-18-mediated IFN-γ release from B19-2D8 cells was assessed. These cells were initially exposed to a high concentration (250 ng/ml) of either the full-length or splice variant of IL-18BP alone. Supernatants from these cultures were unable to induce nitrite production in HD11 cells, demonstrating it is biologically inert (**data not shown**). Next, confirmation of the specific release of IFN-γ by IL-18-stimulated B19-2D8 cells was made, by incubating supernatants from these cells with a polyclonal anti–IFN–γ antibody prior to HD11 stimulation. When compared with IL-18-stimulated B19-2D8 supernatants without this antibody treatment, nitrite production at three different concentrations of IL-18 was significantly lower (Fig. 5A). To determine IL-18 binding protein bioactivity, IL-18 was pre-incubated with either purified full-length or splice variant chIL-18BP for 1 h. Using supernatants from B19-2D8 cells incubated with these IL-18:IL-18BP complexes for 48 h, nitrite production in HD11 cells was inhibited by IL-18BP in a dose-dependent manner (Fig. 5B), when compared with IL-18 only. The intracellular splice variant of chIL-18BP can inhibit chIL-18 as effectively as the full-length protein. Differences in nitrite production between IFN-γ-containing supernatants from chIL-18 + rchIL-18BP groups and the chIL-18 only group were statistically significant at every concentration of rchIL-18BP except for the lowest (15.6 ng/ml).

## 4 Discussion

IL-18 is a potent and fundamentally important cytokine in host defence, particularly against viral infections. In identifying IL-18 in the chicken (Schneider et al., 2000), with bioactivity analogous to its mammalian orthologues (Göbel et al., 2003), it was feasible to expect its endogenous binding protein, IL-18BP, should also exist. Early drafts of the chicken genome did not contain the gene, though a large gap existed in its anticipated position, syntenic with mammalian IL-18BP loci. The year after the initial draft of the chicken genome was published, a truncated chEST provided the first evidence of a chIL-18BP (Watanabe et al., 2005). This paper, however, received little attention and presented no attempt to clone the cDNA or characterise the bioactivity of the protein. Over a decade later, and coinciding with the release of the fifth build of the re-sequenced chicken genome, a study confirmed the genomic location of IL-18BP is conserved in the chicken (Booker and Grattan, 2017). It is important to point out, however, that both genome and article were presenting predicted IL-18BP cDNA and aa sequences derived from uncurated RNA-Seq data. In this study, we present the very first chIL-18BP cDNA sequence derived from multiple molecular clones, which were sequenced using the Sanger chain termination method. A combination of our sequencing data and extensive bioinformatic verification of pre-existing IL-18BP sequences revealed several examples of incorrect cDNA and predicted aa sequences published for this gene. These include the full-length sequences in the Gallus_gallus-5.0 genome build, the sequence published by (Booker and Grattan, 2017), which was derived from this, as well as NCBI Gene records for the mRNA. The current chicken genome build (GRCg6a) has corrected some previous errors, and now presents an mRNA which matches our molecular clone sequences with 100% identity. Despite this, one of the NCBI transcripts (XM_015280901.2) retains an additional error as explained in the results section.

From our bioassays we show both the full-length and splice variant chIL-18BP isoforms inhibit the chIL-18-mediated release of IFN-γ from B19-2D8 cells, confirming the biological function is analogous to mammalian orthologues. The IL-18 binding motif in chIL-18BP has critical conserved residues previously shown to mediate binding between huIL-18BP and huIL-18, which may also be used in the chicken for this purpose. Bioassay data was consistent in all three independent experimental replicates. In humans and mice, a ~10-fold molar excess of IL-18BP is required to fully suppress IL-18 activity (Novick et al., 1999). The highest concentrations of both chIL-18BP variants that were used did not completely abolish IFN-γ production, however, a ~11-fold molar excess of FL and SV led to a 90% and 94% reduction in nitrite production, respectively, indicating a similar ratio of IL-18:IL-18BP is required for complete inhibition in the chicken. Nevertheless, nitrite production was significantly reduced between 62.5 ng/ml and 125 ng/ml chIL-18BP. This specific increase in chIL-18BP increases chIL-18 inhibition from 41 to 72% and 44–71% (SV). These two concentrations of chIL-18BP correspond to molar ratios of 0.72:1 and 1.45:1 (IL-18BP:IL-18). In mammals, IL-18 binds to IL-18BP at an equimolar (1:1) ratio (Novick et al., 2001), which inhibits IL-18 activity by 50–70% (Dinarello, 2001). Our data demonstrates a similar ratio of chIL-18BP:chIL-18 inhibits chIL-18 activity to the same extent. Between 1.45:1 and 10:1 M ratios, only marginally more inhibition was observed. Interestingly, the concentration of nitrites produced at the 1.45:1 M ratio was almost identical to that produced in response to anti–IFN–γ antibody-treated supernatants from IL-18-stimulated B19-2D8 cells when the same concentration of chIL-18 was used.

The existence of a bioactive, intracellular isoform of chIL-18BP is particularly interesting. IL-18BP orthologues in the other species in which we identified a similar splice variant lacking exon 2 and retaining the IL-18 binding motif, although requiring validation, are also likely to be functional. This suggests there may be a specific conserved role for such a splice variant. Intracellular IL-18BP may be required to sequester IL-18 prior to release or during cell death as a safety mechanism; however, alternative functions may also exist. One possibility is regulation of the anti-inflammatory activity of IL-37, as has previously been suggested (Dinarello, 2018). Since IL-37 is known to translocate to the nucleus, one possible function of intracellular IL-18BP may be to bind and sequester this cytokine during inflammatory conditions. Currently, IL-37 has not been identified in any non-mammalian species, suggesting it is unlikely to occur in the chicken. Despite vast quantities of transcriptome and proteome data, it was surprising to find so few species that express this intracellular IL-18BP variant. Further studies are required to adequately understand how broadly conserved and important this variant may be.

When aligned with the predicted chIL-18BP aa isoforms, it was clear which of the two predicted fowlpox viral IL-18BP sequences was likely to bind chIL-18. Our analysis demonstrated fpv214 would be likely to inhibit IL-18 activity in the chicken, whereas fpv073 would theoretically not. Therefore, deleting a vIL-18BP ORF from fowlpox virus, when used as a vaccine vector, should augment the effectiveness of cell-mediated immune responses. Indeed, this appears to be exactly what happens (Skinner et al., 2005), though neither the original data nor a reference to it is available in this review. Furthermore, two highly conserved residues, Y97 and F104, which account for half of the energy required for binding huIL-18BP to huIL-18, are conserved in chIL-18BP (Y71, F78) and fpv247 (Y59, F67). This is also likely to affect the efficacy of any human vaccines using fpv backbones. In support of this prediction, deleting the vIL-18BP (C12L) from modified vaccinia virus Ankara (MVA) leads to significantly enhanced HIV-1-specific T-cell immune responses when MVA is used as a vaccine vector in humans (Falivene et al., 2012). Similarly, vaccinia virus-derived C12L protein inhibits murine IL-18 *in vitro* (Symons et al., 2002). Deleting the vIL-18BP ORF from any avipoxviruses used in humans as vaccine vectors should improve anti-viral host responses.

## Funding

This project was funded by 10.13039/501100000268Biotechnology and Biological Science Research Council (BBSRC) strategic grants to The Pirbright Institute, BBS/E/I/00007031, BBS/E/I/00007034 and BBS/E/I/00007038.

## Figures and Tables

**Supp.Fig. 1:**
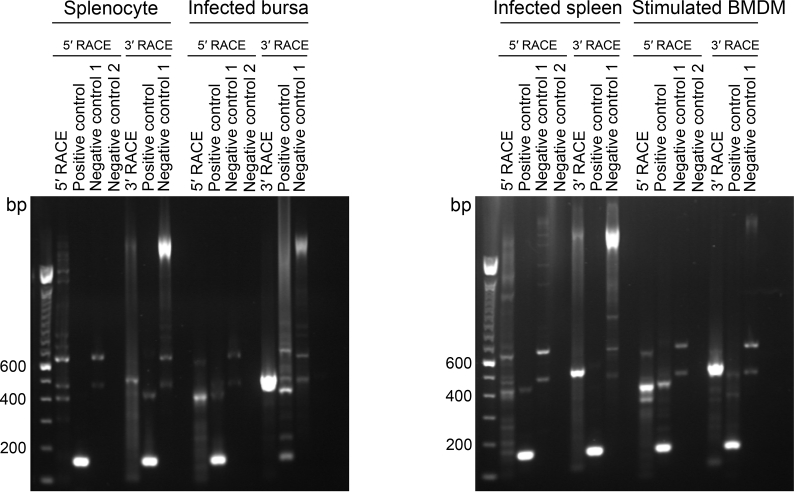
Amplification of full-length and splice variant chIL-18BP cDNAs. 5′ and 3′ rapid amplification of cDNA ends (RACE) was used to amplify FL and SV chIL-18BP cDNAs from infectious bursal disease virus (IBDV)-infected bursa, LPS and CD40L-stimulated bone marrow-derived Line 7 macrophages (BMDM), Salmonella *Typhimurium*-infected RIR splenocyte and unstimulated RIR splenocyte templates. Primers were designed against a putative chIL-18BP EST (I.D: CD727808). Products were separated on a 1% agarose gel and bands of interest were excised, purified and sequenced using the chain termination method.

**Supp.Fig. 2:**
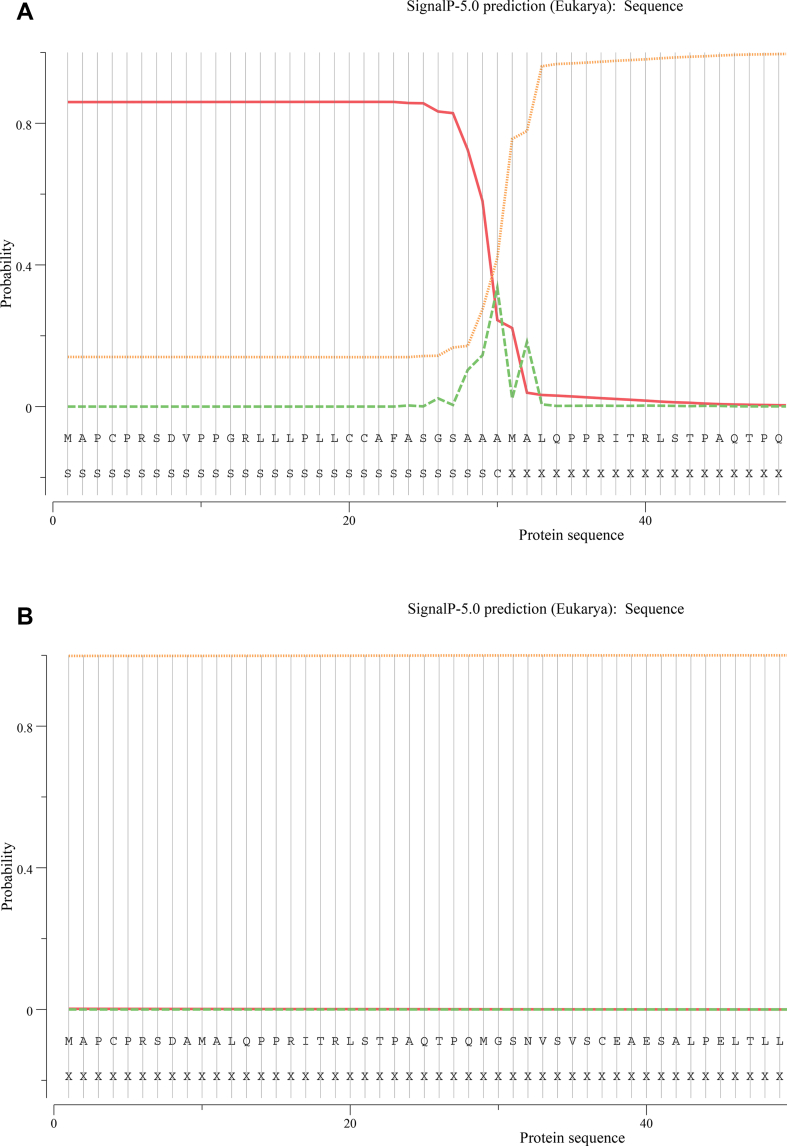
The spliced chIL-18BP amino acid isoform lacks a signal peptide. Predicted FL (A) and SV (B) chIL-18BP aa sequences were analysed for the presence of a signal peptide using the SignalP-5.0 server. In (A), the output plot indicates the FL sequence contains a predicted signal peptide. The red line denotes a signal peptide with high probability spanning the residues, which have the letter S beneath them. The highest peak of the green dotted line indicates the most likely cut site (CS). The yellow dotted sequence (other) does not contain signal peptide. In (B), the absence of red and green dotted lines indicate no signal peptide is predicted.

**Supp.Fig. 3:**
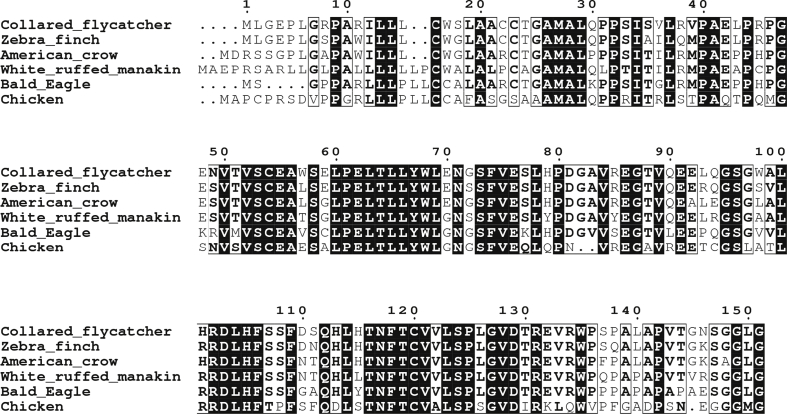
Amino acid alignment of avian IL-18BP sequences. ClustalX was used to align the full-length IL-18BP aa sequences of a group of avian species. These sequences were derived from a BLASTP search of the NCBI database using the full-length chIL-18BP aa sequence. The ALN file derived from the Clustal X alignment was submitted to ESPript 3.0 to create the figure. Residues conserved in all sequences have black background shading. Residues conserved in some of the sequences are in bold and are boxed.
